# Futility of Up-Front Resection for Anatomically Resectable Pancreatic Cancer

**DOI:** 10.1001/jamasurg.2024.2485

**Published:** 2024-07-24

**Authors:** Stefano Crippa, Giuseppe Malleo, Vincenzo Mazzaferro, Serena Langella, Claudio Ricci, Fabio Casciani, Giulio Belfiori, Sara Galati, Vincenzo D’Ambra, Gabriella Lionetto, Alessandro Ferrero, Riccardo Casadei, Giorgio Ercolani, Roberto Salvia, Massimo Falconi, Alessandro Cucchetti

**Affiliations:** 1 Division of Pancreatic Surgery, Pancreas Translational and Clinical Research Center, San Raffaele Scientific Institute, Vita-Salute San Raffaele University, Milan, Italy; 2 Unit of Pancreatic Surgery, Pancreas Institute, University of Verona Hospital Trust, GB Rossi Hospital, Verona, Italy; 3 Department of Oncology and Hemato-Oncology, University of Milano, Italy and HPB Surgery and Liver Transplantation Unit, Fondazione IRCCS Istituto Nazionale Tumori, Milan, Italy; 4 Department of General and Oncological Surgery, Mauriziano Hospital, Turin, Italy; 5 Division of Pancreatic Surgery, IRCCS Azienda Ospedaliero-Universitaria di Bologna, Bologna, Italy; 6 Department of Medical and Surgical Sciences (DIMEC), Alma Mater Studiorum - University of Bologna, Bologna, Italy; 7 Department of Surgery, Morgagni-Pierantoni Hospital, Forlì, Italy

## Abstract

**Importance:**

There are currently no clinically relevant criteria to predict a futile up-front pancreatectomy in patients with anatomically resectable pancreatic ductal adenocarcinoma.

**Objectives:**

To develop a futility risk model using a multi-institutional database and provide unified criteria associated with a futility likelihood below a safety threshold of 20%.

**Design, Setting, and Participants:**

This retrospective study took place from January 2010 through December 2021 at 5 high- or very high-volume centers in Italy. Data were analyzed during April 2024. Participants included consecutive patients undergoing up-front pancreatectomy at the participating institutions.

**Exposure:**

Standard management, per existing guidelines.

**Main Outcomes and Measures:**

The main outcome measure was the rate of futile pancreatectomy, defined as an operation resulting in patient death or disease recurrence within 6 months. Dichotomous criteria were constructed to maintain the futility likelihood below 20%, corresponding to the chance of not receiving postneoadjuvant resection from existing pooled data.

**Results:**

This study included 1426 patients. The median age was 69 (interquartile range, 62-75) years, 759 patients were male (53.2%), and 1076 had head cancer (75.4%). The rate of adjuvant treatment receipt was 73.7%. For the model construction, the study sample was split into a derivation (n = 885) and a validation cohort (n = 541). The rate of futile pancreatectomy was 18.9% (19.2% in the development and 18.6% in the validation cohort). Preoperative variables associated with futile resection were American Society of Anesthesiologists class (95% CI for coefficients, 0.68-0.87), cancer antigen (CA) 19.9 serum levels (95% CI, for coefficients 0.05-0.75), and tumor size (95% CI for coefficients, 0.28-0.46). Three risk groups associated with an escalating likelihood of futile resection, worse pathological features, and worse outcomes were identified. Four discrete conditions (defined as CA 19.9 levels-adjusted-to-size criteria: tumor size less than 2 cm with CA 19.9 levels less than 1000 U/mL; tumor size less than 3 cm with CA 19.9 levels less than 500 U/mL; tumor size less than 4 cm with CA 19.9 levels less than 150 U/mL; and tumor size less than 5 cm with CA 19.9 levels less than 50 U/mL) were associated with a futility likelihood below 20%. Both disease-free survival and overall survival were significantly longer in patients fulfilling the criteria.

**Conclusions and relevance:**

In this study, a preoperative model (MetroPancreas) and dichotomous criteria to determine the risk of futile pancreatectomy were developed. This might help in selecting patients for up-front resection or neoadjuvant therapy.

## Key Points

**Question:**

What are the rates and factors associated with a futile up-front pancreatectomy in patients with anatomically resectable pancreatic ductal adenocarcinoma?

**Findings:**

In this observational study that included 1426 patients, the rate of futile pancreatectomy (death or disease recurrence within 6 months of the operation) was 18.9%. A preoperative model including American Society of Anesthesiologists class, cancer antigen 19.9 levels serum levels, and radiologic tumor size was implemented using a web-based calculator for personalized prediction.

**Meaning:**

These results demonstrate that the ability to predict futile pancreatectomy could help select patients for up-front resection or neoadjuvant therapy.

## Teaser

This study attempts to develop a futility risk model using a multi-institutional database and provide unified criteria associated with a futility likelihood below a safety threshold of 20%.

## Introduction

A combination of tumor resection and systemic treatment is the only chance for long-term survival in patients with pancreatic ductal adenocarcinoma (PDAC).^1^ Current indications for immediate pancreatectomy in surgically fit patients include absence or minimal solid tumor contact with peripancreatic vasculature and no concern for occult metastatic disease.^2^ However, primary resection is associated with a high incidence of postoperative complications with a profound impact on hospital stay, patient recovery, and delay, or even omission of adjuvant chemotherapy.^3,4^ Furthermore, up to 35% of patients have disease recurrence within a year of the operation.^5,6,7,8^ The poor prognostic outlook associated with early recurrence has challenged the effectiveness of pancreatectomy as the primary therapeutic strategy. However, the outcomes from randomized trials of alternative approaches, such as neoadjuvant treatment, have been mixed.^9,10,11,12^ Because randomized trials were not stratified by radiological, biological, or conditional factors, from a real-life perspective, the selection criteria for up-front pancreatectomy or neoadjuvant therapy based on a quantitative estimation of early recurrence risk are ill defined.

The present study aimed to develop a futility risk model for up-front pancreatectomy using a multi-institutional database of patients with anatomically resectable PDAC. In surgical research, an operation is defined as futile when it is not expected to improve the patient’s health, alleviate their symptoms, or prolong life, and when the potential risks and complications outweigh any anticipated benefit.^13,14^ Specifically, we sought to identify pretreatment variables associated with futile pancreatectomy (defined as an operation resulting in patient death or disease recurrence within 6 months) and develop a web-based tool (MetroPancreas) for individual prognostication. Furthermore, unified criteria were searched to maintain the likelihood of a futile pancreatectomy below a safety threshold, weighted against the chance of not receiving postneoadjuvant resection.

## Methods

### Patient Management and Data Collection

Data for patients who underwent up-front pancreatectomy for anatomically resectable PDAC were abstracted from a multi-institutional Italian database, including 2928 consecutive patients who underwent resection between January 2010 and December 2021. Resectability status was defined according to the National Comprehensive Cancer Network (NCCN) and the International Consensus on Definition and Criteria of borderline resectable PDAC, summarized in the eTable in Supplement 1.^2,15^ In resectable disease, a clear fat plane around peripancreatic vasculature with no artery/vein contour irregularity or lumen narrowing was always evident. Exclusion criteria were: (1) anatomic borderline resectable PDAC or locally advanced PDAC, (2) preoperative or intraoperative evidence of metastatic disease, (3) receipt of neoadjuvant therapy, (4) final pathologic diagnosis other than PDAC, (5) incomplete clinical or survival data, and (6) early censoring (less than 6 months postoperatively).

Preoperative staging included a thin-slice, triphasic contrast-enhanced computed tomography of the chest/abdomen within 30 to 40 days before surgery, which was reviewed by dedicated radiologists during multidisciplinary evaluation. External imaging was repeated when considered of poor quality. Although indications for up-front pancreatectomy changed over time and among centers, they were commonly based on the perceived probability of success on the grounds of radiologic features (tumor size, necrotic core, soft tissue stranding), clinical features (symptoms, weight loss, nutritional status), cancer antigen (CA) 19.9 levels, age/life expectancy, and in some cases patient’s preference. Clinical details included demographics, body mass index (BMI), American Society of Anesthesiology (ASA) class, symptoms, tumor markers, radiological staging, and type of resection. Pathologic data included the primary tumor size and grading, the number of harvested and metastatic lymph nodes, the positive to-lymph node ratio, and perineural invasion. Any surgical margin was classified as tumor free if no microscopic tumor was evident within 1 mm of the transection line.^16^

Adjuvant therapy was always considered within 12 weeks of the operation. All patients underwent chest/abdomen contrast-enhanced computed tomography scan and CA 19.9 measurement before adjuvant therapy receipt. The chemotherapy regimens used during the study period varied among participating centers. Most patients received gemcitabine-based chemotherapy with or without radiation therapy. An active radiologic follow-up strategy was performed in all patients with a biannual frequency for the first 2 to 3 years and yearly thereafter. The overall survival (OS) duration was calculated from the time of pancreatectomy until death or last follow-up and the disease-free survival (DFS) was calculated from the time of pancreatectomy until evidence of tumor recurrence or death. Data were locked on October 2023. The study is compliant with Regulation (European Union) 2016/679 of the European Parliament, of the Council of April 27, 2016, and according to Italian law (resolution March 1, 2012, Gazzetta Ufficiale No. 72 of March 26, 2012) on the use and protection of personal data. Ethics approval and informed consent were not required, owing to the retrospective design, the use of anonymized data, and the noninterventional nature of the study. The study is compliant with the Transparent Reporting of a Multivariable Prediction Model for Individual Prognosis or Diagnosis (TRIPOD).^17^

### Outcome Measures

The primary outcome measure was the rate of a futile up-front pancreatectomy. A potentially curative up-front pancreatectomy was defined as futile when death from postoperative complications, PDAC–related events, or cancer recurrence occurred within 6 months of the operation. The reasons for setting a 6-month time mark were multifold. First, we assumed that very early recurrence/death is primarily related to an undetected micrometastatic disease or an incomplete resection.^5,6,7,8^ Second, we assumed that neoadjuvant treatment would have been a viable alternative in case of a futile up-front pancreatectomy.^18^ In this framework, the 6-month time mark corresponds to the immortal time relative to a neoadjuvant therapy course in previous trials and landmark analyses.^19,20^

### Statistical Analysis and Model Development

The overall cohort was split into a derivation cohort including patients from a very high-volume center (Verona), 2 high-volume centers (Bologna and Forlì), an external validation cohort from a very high-volume center (Milan), and a high-volume center (Turin), with a ratio of 62% to 38%. This ratio was set to keep a similar distribution between very high- and high-volume centers within cohorts. In high-volume centers, at least 80 pancreatic resections were performed annually compared with more than 200 resections in very high-volume centers.^21^ Continuous variables were reported as medians with interquartile range (IQR) and were compared using the Mann-Whitney or the Kruskal-Wallis test, as appropriate. Categorical variables were presented as frequencies with percentages and were compared using the χ^2^ or Fisher exact test, as appropriate. Trends were assessed using the Cochran-Armitage test.

A backward logistic regression analysis was performed in the derivation cohort to identify preoperative variables independently associated with futile resection (*P* < .05). Next, a K-fold cross-validation was carried out to correct for overfitting. The number of folds was determined using 20% of the cohort as out-of-sample records of the derivation cohort. Coefficients resulting from the K-fold cross-validation were averaged and discrete risk groups were identified based on tertiles of the linear predictor. The futility risk model was correlated with outcome variables, including histological features, receipt of adjuvant chemotherapy, disease recurrence, and overall survival. Lastly, the model was tested in the external validation cohort and an online calculator was implemented (primary end point). All regression analyses were conducted using robust estimation of standard errors to account for the clustered nature of the multicentric data. Discrimination was assessed through C-statistic^22^^,^ and calibration was evaluated using the Hosmer-Lemeshow test. Specifically, the test calculates if the observed event rates match the expected event rates in population subgroups, with several subgroups set at the number of K-fold cross-validations. A *P* value >.05 at the test indicated that the model is well calibrated.

The secondary end point was to work out unified, dichotomous criteria for surgical candidacy through a simplified version of the algorithm. To this end, a threshold that would allow for a likelihood of a futile up-front resection less than 20% was set under the premise that the harm after a futile up-front pancreatectomy should at least balance the worst scenario of not receiving resection following neoadjuvant therapy. The threshold corresponds to the lower bound of the 95% CI of the postneoadjuvant resection rate from the most recent meta-analyses (resection rate, 0.90; 95% CI, 0.80-1.01; heterogeneity 0%).^23,24^

The OS and DFS curves were constructed using the Kaplan-Meier method and pairwise differences between groups were assessed using the log-rank test. All the analyses were conducted using Stata version 18.0 (StataCorp).

## Results

A total of 1426 patients met the inclusion criteria and were included in the analysis. The study flowchart is shown in the eFigure in Supplement 1. The median age was 69 (IQR, 62-75) years. Most patients were male (759 [53.2%]) with ASA class I or II (860 [60.6%]). Pancreatoduodenectomy was the most common procedure (945 [66.3%]) and 1051 patients received adjuvant treatment (73.7%). Within 6 months of pancreatectomy, tumor recurrence was diagnosed in 199 patients (14.0%), 97 patients died of postoperative complications or other causes (6.8%), and 27 (1.8%) died of disease. Therefore, pancreatectomy proved to be futile in 269 patients (18.9%). In the overall cohort, the median follow-up was 25.2 (IQR, 14.3-44.1) months, the median DFS was 16.2 months (95% CI, 15.1-17-4), and the median OS was 34.1 months (95% CI, 32.2-37.1).

The derivation cohort included 885 patients and the validation cohort included 541 patients. While most of the preoperative clinical features were significantly different between the 2 cohorts, oncologic outcomes, including DFS and OS, were comparable (Table 1).

**Table 1.** Preoperative Clinical Characteristics and Outcome Measures of Derivation and Validation Cohorts After Up-Front Surgery of Anatomically Resectable Pancreatic Ductal Adenocarcinoma

Characteristic	Cohort, No. (%)		*P* value
	Derivation (n = 885)^a^	Validation (n = 541)	
Clinical features			
Age, y, median (IQR)	68 (61-74)	71 (64-76)	.001
Sex			
Female	419 (47.3)	249 (45.8)	.31
Male	466 (52.7)	293 (54.2%)	
Symptoms at diagnosis			
Pain	128 (14.5)	101 (18.7)	.02
Weight loss	397 (44.9)	184 (34.0)	.001
Jaundice	496 (56.1)	294 (54.3)	.28
Head location	649 (73.3)	427 (78.9)	.01
ASA class			
I	29 (3.3)	20 (3.7)	.01
II	535 (60.5)	280 (51.8)	
III	321 (36.3)	241 (44.6)	
Radiological size, median (IQR), cm	2.7 (2.0-3.1)	2.5 (2.0-3.0)	.04
CA 19-9 level, median (IQR), U/mL	93 (26-249)	47 (3-199)	.001
Log CA 19-9 level, median (IQR), U/mL	1.96 (1.41- 2.39)	1.76 (0.48-2.29)	.001
Type of operation			
Pancreatoduodenectomy	552 (62.4)	393 (72.6)	.001
Distal pancreatectomy	218 (24.6)	107 (19.8)	
Total pancreatectomy	115 (13.0)	41 (7.6)	
Adjuvant treatment	672 (75.9)	379 (70.1)	.02
Outcomes			
30-d Mortality	12 (1.4)	11 (2.0)	.39
90-d Mortality	39 (4.4)	22 (4.1)	.79
Death within 6 mo	56 (6.3)	41 (7.6)	.39
Recurrence within 6 mo	117 (13.2)	82 (15.2)	.30
Futility of up-front surgery^b^	165 (18.6)	104 (19.2)	.78
DFS median (95% CI), mo	16.5 (15.0-18.1)	15.9 (14.3-17.8)	.96
OS, median (95% CI), mo	35.1 (32.1-37.4)	33.7 (28.8-38.6)	.45
Follow-up length, median (IQR)	24.8 (14.2-44.3)	25.8 (14.7-44.1)	.81

Abbreviations: ASA, American Society of Anesthesiology; CA, cancer antigen; DFS, disease-free survival; IQR, interquartile range; OS, overall survival.

^a^ The derivation cohort was formed by 3 very high-volume centers (Verona) and 2 high-volume centers (Bologna and Forlì). The external validation cohort was formed by 1 very high-volume center (Milan) and 1 high-volume center (Turin).

^b^ Defined as death or recurrence within 6 months of resection. Twenty-seven patients (8 in the derivation cohort and 19 in the validation cohort) had recurrence/died within this time frame.

### Futility Risk Model

The preliminary regression model identified ASA class (95% CI for coefficients, 0.68-0.87; *P* = .001), preoperative CA 19.9 serum (95% CI for coefficients, 0.05-0.75; *P* = .04), and radiological tumor size (95% CI for coefficients, 0.28-0.46; *P* = .001) as the most robust independent variables associated with futile pancreatectomy. These 3 variables entered the subsequent K-fold cross-validation regression (Table 2). After averaging variable coefficients, the C-statistic of the out-of-sample cohorts was 0.68 (95% CI, 0.63-0.72), indicating good discrimination and likely avoiding model overfit. The Hosmer-Lemeshow test *P* value was 0.39, indicating adequate calibration. An individual case prognostication algorithm (MetroPancreas) is available online.^25^

**Table 2.** Modeling Futility (Recurrence or Death Within 6 Months) After Up-Front Surgery of Anatomically Resectable Pancreatic Ductal Adenocarcinoma Based on Preoperative Clinical Features in the Derivation Cohort

	k-Fold cross validation					Pooled (95% CI)
	1	2	3	4	5	
In-sample cohorts, No.	708	708	708	708	708	885
Coefficients (95% CI)						
ASA class III	0.906 (0.805-1.006)	0.681 (0.603-0.759)	0.687 (0.575-0.798)	0.781 (0.647-0.915)	0.841 (0.803-0.880)	0.779 (0.687-0.871)
Tumor size, cm	0.399 (0.301-0.496)	0.384 (0.309-0.459)	0.334 (0.250-0.417)	0.424 (0.391-0.458)	0.332 (0.148 to 0.517)	0.375 (0.280-0.469)
Ca 19-9 level (per log10)	0.450 (0.009-0.892)	0.251 (0.169-0.671)	0.409 (0.003-0.814)	0.389 (0.016-0.763)	0.392 (0.148 to 0.636)	0.378 (0.005-0.751)
Constant value	−3.775 (−0.485 to −2.901)	−3.335 (−4.233 to −2.437)	−3.493 (−4.422 to −2.564)	−3.788 (−4.944 to −2.632)	−3.524 (−3.781 to −3.266)	−3.603 (−4.442 to −2.764)
C-statistic (95% CI)	0.706 (0.657-0.754)	0.667 (0.617-0.716)	0.671 (0.621-0.720)	0.690 (0.640-0.740)	0.673 (0.624-0.723)	NA
Hosmer-Lemeshow, *P* value	.32	.33	.26	.28	.26	NA
Out-of-sample cohorts, No.	177	177	177	177	177	885
C-statistic (95% CI)	0.568 (0.465-0.672)	0.739 (0.645-0.834)	0.720 (0.620-0.820)	0.644 (0.546-0.742)	0.706 (0.608-0.803)	0.681 (0.636-0.725)
Hosmer-Lemeshow, *P* value^a^	.04	.70	.83	.17	.60	.39

Abbreviations: ASA, American Society of Anesthesiology; NA, not applicable.

^a^ The Hosmer-Lemeshow *P* > .05 indicated that the model fits reasonably well. The model’s linear predictor can be calculated as follows: 0.799 if ASA 3 + 0.375 × tumor size in cm + 0.378 × log10 of CA 19-9 level of –3.603. The linear predictors’ tertiles were quarter 1, less than −1.961; quarter 2, between −1.961 and – 1.331; and quarter 3, less than −1.331. The probability of futility can be calculated as 1 / (1 + EXP [ − linear predictor]).

**Figure 1.**
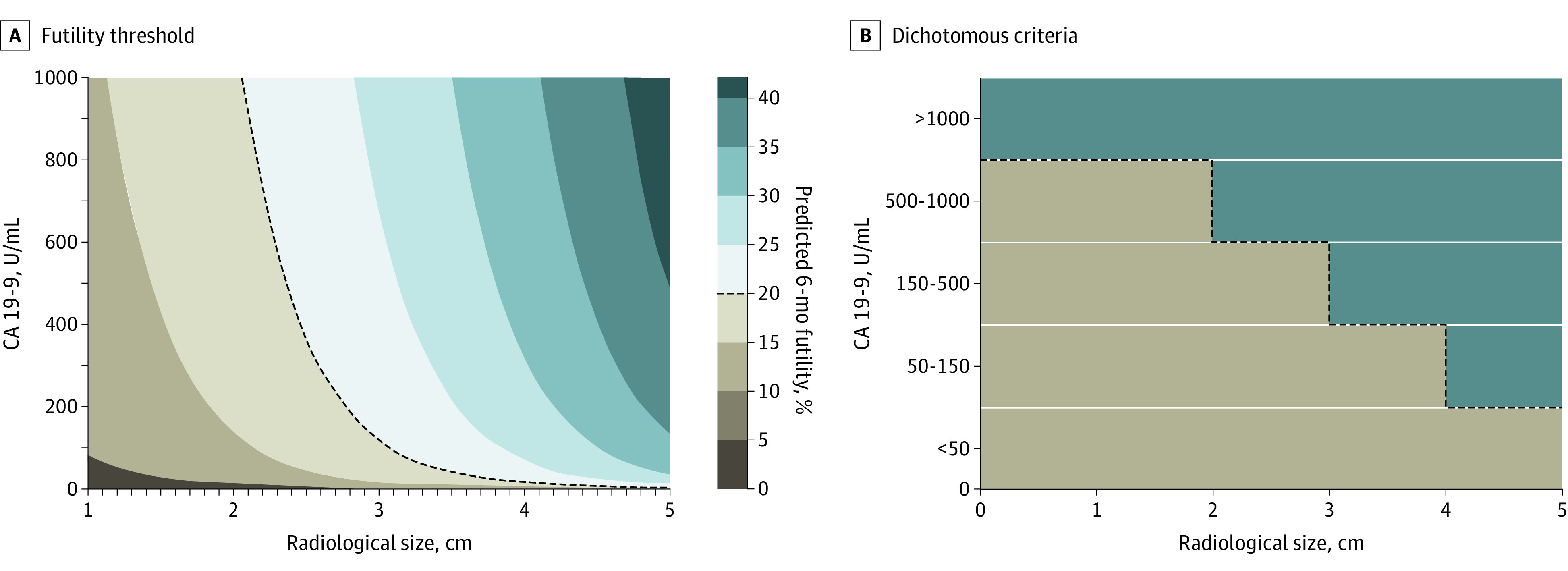
Contour Plot Outlining the Likelihood of Futile Up-Front Pancreatectomy as a Function of Tumor Size and Serum Carbohydrate Antigen (CA) 19.9 The dotted line represents the futility threshold, set at 20% (A). Dichotomous (in/out) criteria associated with a likelihood of futile pancreatectomy below the 20% threshold (B).

Three risk groups (low, intermediate, and high) were identified based on tertiles of the model linear predictor. Lastly, applying the model in the external validation cohort was associated with a C-statistic of 0.65 (95% CI, 0.60-0.70) and a Hosmer-Lemeshow *P* = .19, indicating adequate discrimination and calibration. In the derivation cohort, the rate of futile pancreatectomy was 9.2% in the low-risk group, 18.0% in the intermediate-risk group, and 28.7% in the high-risk group, respectively (*P* < .001 for trend). In the validation cohort, the futility rate was 10.9% in the low-risk group, 20.2% in the intermediate-risk group, and 29.2% in the high-risk group (*P* < .001 for trend). In both cohorts, the risk groups were also associated with an escalating likelihood of adverse pathologic features and with a sharp decrease in DFS and OS durations (Table 3**)**. Figure 1 summarizes, in a contour plot, variations in the likelihood of a futile pancreatectomy as a function of independent predictors (radiological tumor size on the x-axis and preoperative CA 19.9 serum levels on the y-axis, at the mean of the ASA III class in the whole cohort).

**Table 3.** Futility, Pathological Features, Likelihood of Adjuvant Therapy Receipt, and Oncologic Outcomes Stratified by Risk Classes in the Derivation and Validation Cohorts

Variable	Risk, No. (%)			*P* value for trend
	Low	Intermediate	High	
Derivation cohort, No.	295	295	295	NA
Histology				
Grade 3-grade 4	77 (26.1)	78 (26.8)	82 (27.8)	.04
Node-positive	212 (71.9)	244 (82.7)	255 (86.4)	.001
PLNR >20%	51 (17.3)	67 (22.7)	83 (28.1)	.002
R0	191 (64.8)	164 (55.6)	146 (49.5)	.001
Adjuvant chemotherapy	250 (84.8)	223 (75.6)	199 (67.5)	.001
Futility	27 (9.2)	53 (18.0)	85 (28.7)	.001
Death	7 (2.4)	17 (5.8)	32 (10.9)	.001
Recurrence	20 (6.8)	36 (12.2)	61 (20.7)	.001
DFS, median (95% CI), mo	24.0 (18.8-28.2)	15.6 (13.7-18.5)	12.3 (10.6-14.4)	.001
OS, median (95% CI), mo	50.2 (40.8-61.3)	33.2 (27.5-37.4)	24.4 (21.4-27.8)	.001
Validation cohort, No.	212	168	161	NA
Histology				
Grade 3-grade 4	103 (48.6)	105 (62.5)	112 (69.6)	.001
Node-positive	154 (72.6)	136 (81.0)	135 (83.9)	.01
PLNR >20%	42 (19.8)	48 (28.6)	48 (29.8)	.02
R0	142 (67.0)	100 (59.5)	91 (56.5)	.04
Adjuvant chemotherapy	161 (75.9)	112 (66.7)	106 (65.8)	.03
Futility	23 (10.9)	34 (20.2)	47 (29.2)	.001
Death	8 (3.8)	12 (7.1)	21 (13.0)	.001
Recurrence	18 (8.5)	27 (16.1)	37 (23.0)	.001
DFS, median (95% CI), mo	24.0 (18.2-32.6)	14.0 (11.2-16.5)	12.3 (9.5-15.1)	.001
OS, median (95% CI), mo	51.0 (40.3-66.8)	27.7 (24.1-32.4)	23.9 (19.2-28.8)	.001

Abbreviations: DFS, disease-free survival; NA, not applicable; OS, overall survival; PNLR, positive to lymph node ratio; R0, no residual tumor.

**Figure 2.**
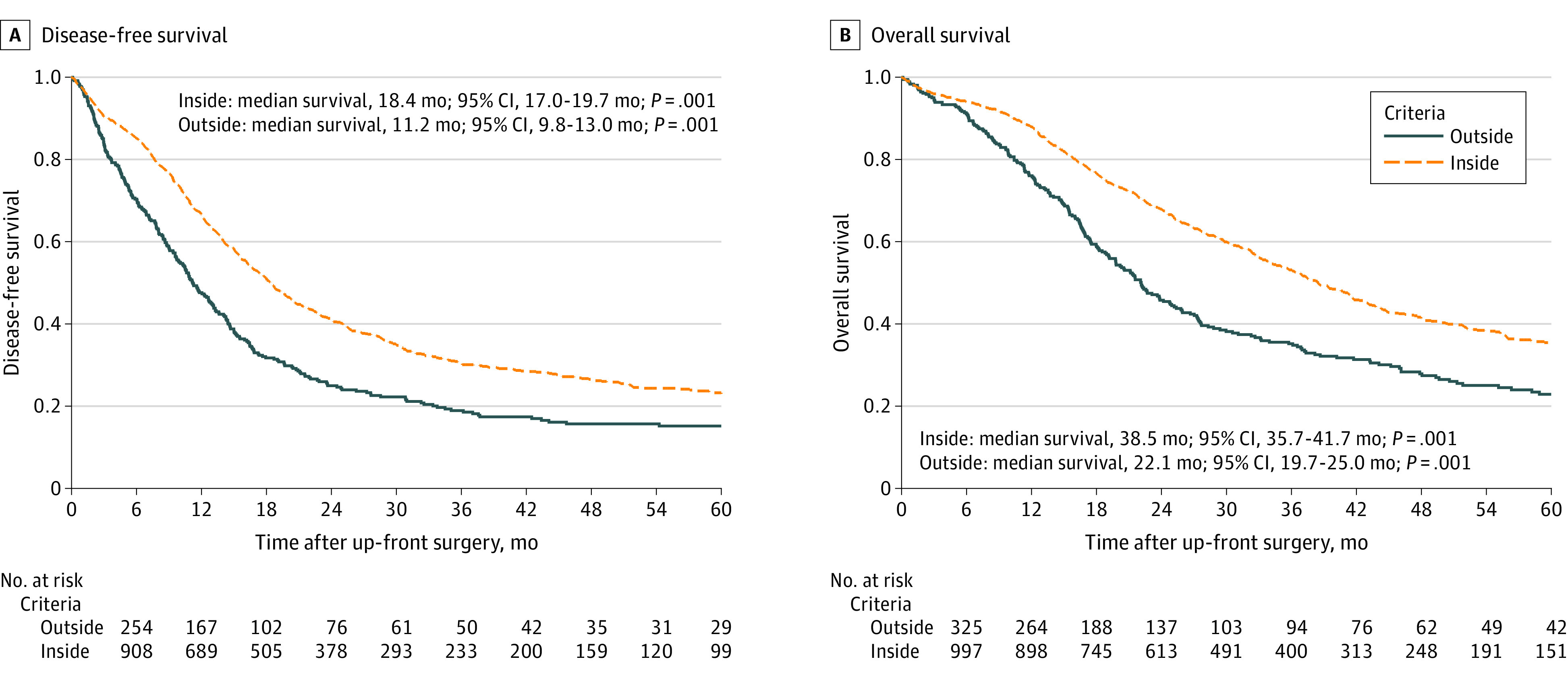
Disease-Free Survival and Overall Survival After Up-Front Resection in Patients Who Fulfilled the Cancer Antigen 19-9-Adjusted-to-Size Criteria and Those Who Did Not

Another end point was to provide unified, dichotomous criteria for treatment allocation that would allow for a likelihood of a futile up-front resection less than 20%. This was satisfied in 4 preoperative conditions, defining the CA 19-9-adjusted-to-size criteria, summarized in Figure 1: (1) tumor size less than 2 cm with CA 19.9 levels less than 1000 U/mL, (2) tumor size less than 3 cm with CA 19.9 levels less than 500 U/mL, (3) tumor size less than 4 cm with CA 19.9 levels less than 150 U/mL, and (4) tumor size less than 5 cm with CA 19.9 levels less than 50 U/mL. In the study cohort, 1065 of 1426 patients (74.7%) met these criteria with an overall rate of futile pancreatectomy approaching 15% (159 patients). The upper 99% confidence limit was 18.0%, within the initial threshold set at 20%. As outlined in Figure 2, the DFS was considerably worse in patients who did not fulfill the CA 19.9-adjusted-to-size criteria (median DFS of 11.2 months; 95% CI, 9.8-13.0 vs 18.4 months; 95% CI, 17.0-19.7; *P* = .001). The median OS of patients within the CA 19-9-adjusted-to-size criteria was 38.5 months (95% CI, 35.7-41.7) compared with 22.1 months (95% CI, 19.7-25.0) in patients not fulfilling these criteria (*P* = .001; Figure 2B). Data on the association between futile pancreatectomy, tumor site, and receipt of adjuvant therapy are provided in the eResults in Supplement 1. Detailed outcomes of patients outside the CA 19-9-adjusted-to-size criteria are provided in the eResults in Supplement 1.

## Discussion

The primary treatment of anatomically resectable PDAC is a matter of controversy. While the 2023 European Society for Medical Oncology guidelines endorse a surgery-first approach,^26^ the NCCN guidelines (version 1.2024) recommend either up-front resection (in the absence of risk features for occult metastatic disease) or neoadjuvant treatment (regardless of the presence or absence of risk features).^2^ Nonetheless, the concept of high risk is mainly qualitative, as the definition includes elevated CA 19.9 serum levels, large primary tumor, suspicion of nodal metastases on imaging, excessive weight loss, and significant pain.^2^ Despite the derivation of different CA 19.9 serum levels thresholds (1000 U/mL per the MD Anderson Cancer Center criteria and 500 U/mL per the International Association of Pancreatology criteria)^15,27^ or the application of anatomy/biology/conditional classification for enucleating patients with so-called biologic borderline-resectable PDAC,^28^ a clear-cut definition of high-risk resectable disease remains undetermined.

In the present analysis, we investigated the pretreatment variables associated with a futile primary resection (death for postoperative complications, PDAC–related events, or disease recurrence within 6 months postoperatively) and implemented a risk model for futility. The 6-month time mark corresponds to the definition of very early recurrence,^7^ although the cutoffs used throughout the present literature to classify PDAC recurrence as very early or early are arbitrary or defined as the point with the lowest log-rank *P* value for overall and postrecurrence survival function split.^5,6,7,8,29,30^ Despite these methodology issues, it is biologically likely that a recurrence within 6 months of pancreatectomy was due to undetected micro-metastatic disease at baseline or incomplete resection. Furthermore, we assumed that neoadjuvant treatment would have been viable in patients at high risk for futile up-front pancreatectomy.^23^ In this framework, the 6-month time mark fits with the immortal time associated with a neoadjuvant therapy course in previous landmark analyses and clinical trials, where the period between baseline evaluation and resection ranged from 3.5 to 7 months.^19,20,31^

The main variables associated with futile pancreatectomy were ASA class, preoperative CA 19.9 serum, and radiological tumor size. The resulting model identified 3 risk categories based on the linear predictor tertiles, which not only were associated with futility, but also with adverse pathological features, receipt of adjuvant therapy, recurrence rates, and OS in both cohorts. An online calculator (MetroPancreas) was implemented to quantify the likelihood of futility and assign a risk class to any individual patient. Remarkably, the model showed adequate discrimination and very good calibration in the out-of-sample and validation cohorts despite substantial baseline differences relative to the derivation cohort. This was due to the 5-fold cross-validation approach, which reduced the model over optimism in the derivation cohort, improving its generalizability.

Furthermore, the concept of futile pancreatectomy as a function of radiological tumor size and baseline CA 19.9 levels was simplified, identifying 4 discrete conditions (defined as CA 19.9-adjusted-to-size criteria) that maintain the likelihood of a futile pancreatectomy below 20%. This safety threshold was set under the premise that the harm of a futile pancreatectomy should be equal to the chance of not receiving postneoadjuvant resection, expressed as the lower bound of the 95% CI for resection rates in published meta-analyses (0.80).^23,24^

Both the DFS and the OS duration of patients fulfilling the criteria (tumor size less than 2 cm with CA 19.9 levels less than 1000 U/mL, tumor size less than 3 cm with CA 19.9 levels less than 500 U/mL, tumor size less than 4 cm with CA 19.9 levels less than 150 U/mL, or tumor size less than 5 cm with CA 19.9 levels less than 50 U/mL) were significantly longer than patients outside the criteria. While preoperative tumor size and CA 19.9 serum levels have been variously associated with survival, their impact was primarily investigated in isolation.^32,33,34,35,36^ The few available prognostic scores based on pretreatment variables were closely associated with OS. Still, they lacked information on early recurrence and were not constructed to identify patients at risk for futile procedures.^37,38^

The methodological approach adopted here stems from the liver transplant oncology experience some of us had. Clinical criteria for transplant eligibility of patients with hepatocellular carcinoma have been available since 1996^39^ and refined to the more recent flexible Metroticket 2.0 model concept.^40^ In the context of hepatocellular carcinoma, eligibility criteria for transplant are necessary due to organ shortage. In PDAC, eligibility criteria can be used during baseline assessment to limit surgical futility and redirect to neoadjuvant therapy patients at the highest risk of early recurrence or death.

### Limitations

This study also has significant limitations, primarily related to its retrospective design. Preoperative imaging was not revised with possible variability in the assignment of resectability classes per the NCCN criteria.^41^ Furthermore, there were institutional differences in the selection process for up-front pancreatectomy and not all patients presenting with jaundice underwent preoperative biliary drainage. In those who did not receive biliary drainage, hyperbilirubinemia may have altered CA 19.9 serum levels with possible biases to the model. The model cannot be applied to CA 19.9 nonsecretors, who account for 10% of patients with PDAC. Also, all types of pancreatic resection were analyzed together. While tumor location was not associated with futile pancreatectomy in sensitivity analysis and did not affect outcomes in previous studies,^42^ differences in the model performance could exist when stratifying by head vs body-tail tumors. Furthermore, the overall discrimination was only adequate (C-index of 0.68 in the derivation cohort and 0.65 in the validation cohort). Integration with other variables not included in the present dataset (ie, radiomic signatures) and machine learning approaches could improve the prediction ability in future efforts.^43^ Lastly, the model was not externally validated. Additional validation from other health care systems (ie, Eastern or US patient cohorts) would improve its global uptake.

## Conclusions

Considering these limitations, the present study provided a prognostic model to identify patients with anatomically resectable PDAC who are unlikely to benefit from up-front pancreatectomy because of death or tumor recurrence within 6 months of the operation. The likelihood of a futile resection was modeled as a function of ASA class, preoperative tumor size, and CA 19.9 levels to construct a readily available online risk calculator (MetroPancreas).^25^ Furthermore, unified CA 19.9-adjusted-to-size criteria were provided, which can further help in the triaging process and trial design, assuming that neoadjuvant therapy would be a viable option when the criteria are unmet.

## Notes

Supplement 1. eTable. Criteria defining resectability status at diagnosis eFigure. Study flowchart eResults. Role of adjuvant treatment; location and futility; outcomes of patients at high risk of futile resection

Supplement 2. Data sharing statement
