# Supplementary material for: Futility of Up-Front Resection for Anatomically Resectable Pancreatic Cancer
Source: JAMA Surg. 2024 Jul 24;159(10):1139–47. doi: 10.1001/jamasurg.2024.2485 (PMC11270270; doi:10.1001/jamasurg.2024.2485)
Supplement: Supplement 1. — eTable. Criteria defining resectability status at diagnosis eFigure. Study flowchart eResults. Role of adjuvant treatment; location and futility; outcomes of patients at high risk of futile resection [file jamasurg-e242485-s001.pdf]

Supplemental Online Content

Crippa S, Malleo G, Mazzaferro V, et al. Futility of upfront resection for anatomically resectable pancreatic cancer. *JAMA Surg*. Published online July 24, 2024. doi:10.1001/jamasurg.2024.2485

eTable. Criteria defining resectability status at diagnosis

eFigure. Study flowchart

eResults. Role of adjuvant treatment; location and futility; outcomes of patients at high risk of futile resection

This supplemental material has been provided by the authors to give readers additional information about their work.

eTable 1. Criteria defining resectability status at diagnosis.

© 2024 [First author last name and initials or group name] et al. *[Journal name].*

**Resectability Status**	**Arterial**	**Venous**
**Resectable**	• No arterial tumor contact (celiac axis [CA], superior mesenteric artery [SMA], or common hepatic artery [CHA]).	• No tumor contact with the superior mesenteric vein (SMV) or portal vein (PV) or ≤180° contact without vein contour irregularity.
**Borderline resectable**	**Pancreatic head/uncinate process:**<br>• Solid tumor contact with CHA without extension to CA or hepatic artery bifurcation allowing for safe and complete resection and reconstruction.<br>• Solid tumor contact with the SMA of ≤180°.<br>• Solid tumor contact with variant arterial anatomy (eg, accessory right hepatic artery, replaced right hepatic artery, replaced CHA, and the origin of replaced or accessory artery) and the presence and degree of tumor contact should be noted if present, as it may affect surgical planning.<br>**Pancreatic body/tail:**<br>• Solid tumor contact with the CA of ≤180°.	• Solid tumor contact with the SMV or PV of >180°, contact of ≤180° with contour irregularity of the vein or thrombosis of the vein but with suitable vessel proximal and distal to the site of involvement allowing for safe and complete resection and vein reconstruction.<br>• Solid tumor contact with the inferior vena cava (IVC).
**Locally advanced**	**Head/uncinate process:**<br>• Solid tumor contact >180° with the SMA or CA.<br>**Pancreatic body/tail:**<br>• Solid tumor contact of >180° with the SMA or CA.<br>• Solid tumor contact with the CA and aortic involvement.	• Unreconstructible SMV/PV due to tumor involvement or occlusion (can be due to tumor or bland thrombus).

Adapted from NCCN Clinical Practice Guidelines in Oncology (NCCN Guidelines®), Pancreatic Adenocarcinoma, Version 1.2024 — December 13, 2023. www.NCCN.org. Accessed on April 27th, 2024.

eFigure 1. Study flowchart. Data were abstracted from a joint database of five Italian institutions (three high-volume centers, at least 80 pancreatic resections/year, and two very-high-volume centers, at least 200 pancreatic resections/year).

© 2024 [First author last name and initials or group name] et al. *[Journal name].*

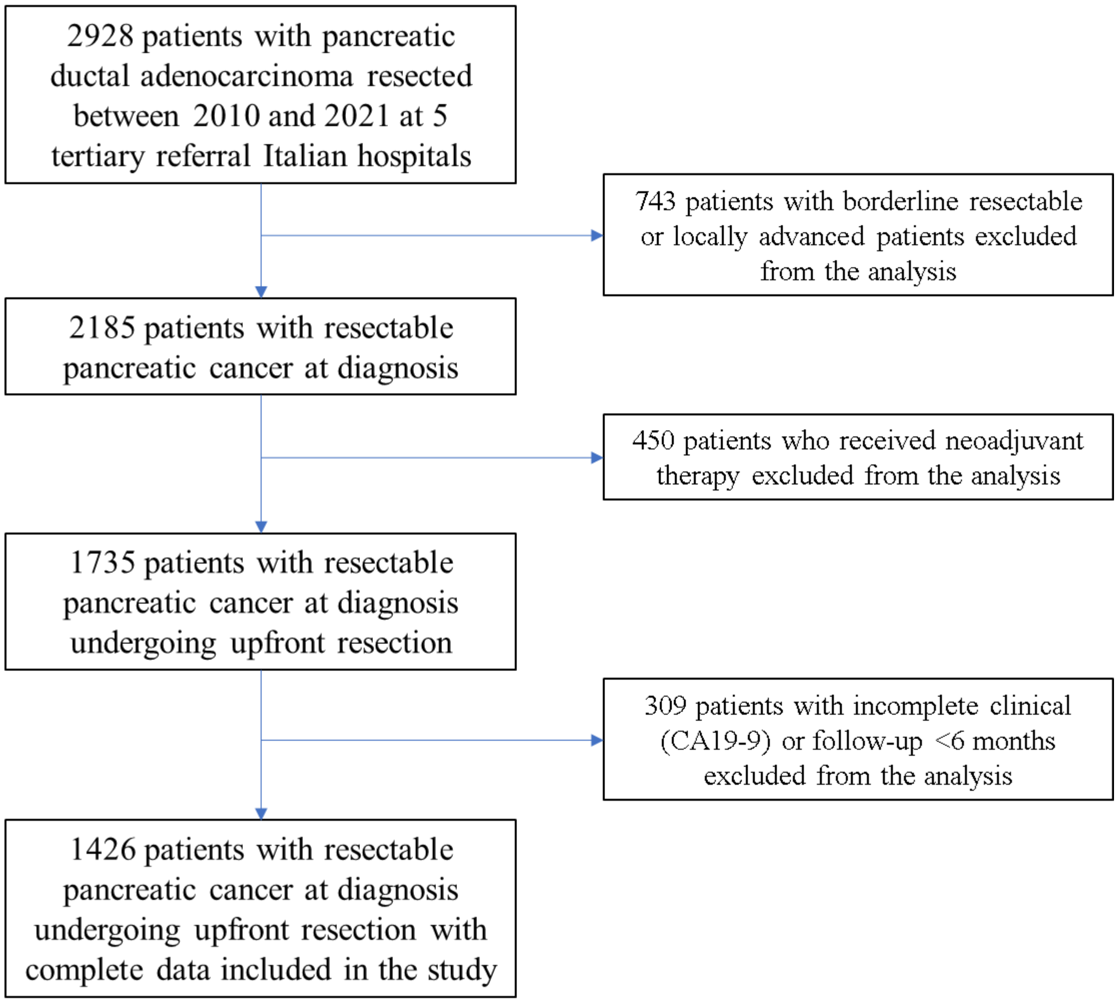

eResults

Role of adjuvant treatment

© 2024 [First author last name and initials or group name] et al. *[Journal name].*

In the group that did not match the CA19-9-adjusted-to-size criteria, the rate of futile pancreatectomy was 20.9% (95%CI 16.0-26.5) in patients who received adjuvant therapy and 52.8% (95%CI 42.2-61.3) in those patients who did not (or died because of post-operative complications).
In the group matching the CA19-9-adjusted-to-size criteria, the rate of futile pancreatectomy was 8.1% (95%CI 6.3-10.2) in patients who received adjuvant therapy and 35.7% (95%CI 29.9-41.9) when adjuvant therapy was omitted. The rate of adjuvant therapy receipt was 80.6% (95%CI 77.8-83.1) in ASA I-II patients and 63.2% (95%CI 59.0-67.7) in ASA III-IV patients.

Tumor location and futility
In the group that did not match the CA19-9-adjusted-to-size criteria, the rate of futile resection was 30.7% (95%CI 24.6-37.3) in patients undergoing distal pancreatectomy and 30.1% (95%CI 22.8-38.3) in those who underwent pancreatoduodenectomy or total pancreatectomy.
In the group matching the CA19-9-adjusted-to-size criteria, the rate of futile resection was 14.3% (95%CI 11.8-17.0) in patients undergoing distal pancreatectomy and 16.4% (95%CI 12.6-20.8) in those who underwent pancreatoduodenectomy or total pancreatectomy. Both p-values were >0.1, thus, tumor location was not associated with a different rate of futile resection.

Outcomes of patients at high risk of futile resection
A total of 361 patients were outside the CA19-9-adjusted-to-size *criteria* (thus, at high risk for futile pancreatectomy). Among them, 251 patients survived beyond the 6-month threshold. One hundred and thirty-five patients (53.8%) subsequently died of disease, 35 (13.9%) are alive with recurrence, 16 (6.4%) died of other causes, and 65 (25.9%) are alive and disease-free. Therefore, the ratio between patients alive and disease-free and the 6-month mark and the population-at-risk was 18.0% (65/361) This latter figure in patients matching the CA19-9-adjusted-to-size criteria (thus, at low risk for futile pancreatectomy) was 34.9% (316/906), further confirming the utility of the model.

© 2024 [First author last name and initials or group name] et al. *[Journal name].*
