# Supplementary material for: Futility of Up-Front Resection for Anatomically Resectable Pancreatic Cancer
Source: JAMA Surg. 2024 Jul 24;159(10):1139–47. doi: 10.1001/jamasurg.2024.2485 (PMC11270270; doi:10.1001/jamasurg.2024.2485)
Supplement: Supplement 2. — Data sharing statement [file jamasurg-e242485-s002.pdf]

Data Sharing Statement

Crippa. Futility of Up-Front Resection for Anatomically Resectable Pancreatic Cancer. *JAMA Surg*. Published July 24, 2024. doi:10.1001/jamasurg.2024.2485

Data
Data available: Yes
Data types: Other (please specify)
Additional Information: Anonymized data
How to access data: Anonymized data will be shared by specific requests to the corresponding author: vincenzo.mazzaferro@istitutotumori.mi.it
When available: With publication

Supporting Documents
Document types: None

Additional Information
Who can access the data: Researchers whose proposed use of the data has been approved
Types of analyses: For any purpose
Mechanisms of data availability: Data will be available after approval of a proposal
